# Influence of electrolyte co-additives on the performance of dye-sensitized solar cells

**DOI:** 10.1186/1556-276X-6-307

**Published:** 2011-04-07

**Authors:** Thomas Stergiopoulos, Evangelia Rozi, Chaido-Stefania Karagianni, Polycarpos Falaras

**Affiliations:** 1 Institute of Physical Chemistry, NCSR "Demokritos", Aghia Paraskevi Attikis, Athens 15310, Greece; 2 School of Chemical Engineering, National Technical University of Athens, 9 Iroon Polytechniou St., 15780 Zografou, Athens, Greece

## Abstract

The presence of specific chemical additives in the redox electrolyte results in an efficient increase of the photovoltaic performance of dye-sensitized solar cells (DSCs). The most effective additives are 4-*tert*-butylpyridine (TBP), *N*-methylbenzimidazole (NMBI) and guanidinium thiocyanate (GuNCS) that are adsorbed onto the photoelectrode/electrolyte interface, thus shifting the semiconductor's conduction band edge and preventing recombination with triiodides. In a comparative work, we investigated in detail the action of TBP and NMBI additives in ionic liquid-based redox electrolytes with varying iodine concentrations, in order to extract the optimum additive/I_2 _ratio for each system. Different optimum additive/I_2 _ratios were determined for TBP and NMBI, despite the fact that both generally work in a similar way. Further addition of GuNCS in the optimized electrolytic media causes significant synergistic effects, the action of GuNCS being strongly influenced by the nature of the corresponding co-additive. Under the best operation conditions, power conversion efficiencies as high as 8% were obtained.

## Introduction

Current efficiencies of dye-sensitized solar cells (DSCs) can compete with the ones gained by established photovoltaic systems such as monocrystalline Si [1]. The DSC main feature is its photoelectro-chemical nature, meaning that the efficiency can be optimized by varying the chemical composition of its components. Efficiency increase can be achieved, for instance, by modifying the light absorbing antenna [2]. In another, much easier approach, optimization of the electrolyte would also lead to increased device efficiency [3-5].

The most convenient way to enhance the photovoltaic efficiency is the addition of appropriate chemical species in the electrolyte to fine tune the semiconductor-electrolyte interface. For instance, nitrogen heterocyclic compounds such as 4-*tert*-butylpyridine (TBP) and *N*-methylbenzimidazole (NMBI) are added in the electrolyte to improve the open-circuit potential (*V*_oc_) [6,7] while guanidinium thiocyanate (GuNCS) was found to increase both *V*_oc _and the short-circuit photocurrent (*J*_sc_) [8,9]. The exact role of the additives is now well known; it seems that TBP and NMBI deprotonate the TiO_2 _surface by adsorption and thus shift the conduction band edge (*E*_c_) toward negative potentials and passivate the surface active recombination sites [10,11]. On the contrary, GuNCS species accumulate their positive charge on the semiconductor surface, inducing a positive shift of the *E*_c_, thus increasing the electron injection efficiency [12] and simultaneously slowing down recombination at open-circuit conditions [8].

Despite the fact that a few papers have already dealt with the exact role of each additive separately, no other work has ever presented the synergistic effects of these additives in a single identical system and no comparison between these effects has ever been made. Furthermore, although it is well known that these additives affect directly recombination reaction with the iodine, the effects of varying I_2 _concentration need further investigation [13]. Thus, in this work, we comparatively studied the effects of TBP and NMBI additives in redox electrolytes of varied iodine concentration. When optimum TBP/I_2 _and NMBI/I_2 _concentration ratios were determined, the effects of the addition of GuNCS in these systems were further investigated.

## Experimental

### Preparation of electrolytes

Ionic liquid (IL)-based electrolytes were prepared by adding 0.8 M of 1-methyl-3-propylimidazolium iodide (PMII, Fluka) in propylene carbonate (PC, Fluka). PC solvent is used in DSCs for its high dielectric constant [14,15]. Keeping the PMII composition stable, we examined the effect of the I_2 _concentration by adding different quantities (0.02-0.08 M) of iodine (resublimed I_2_, Aldrich) in the above mixture. Further addition of NMBI or TBP (Aldrich) was performed. When the optimum NMBI or TBP-based electrolyte compositions (0.45 M) were determined (respectively), 0.05 M of GuNCS (Aldrich) was added in each system [16,17]. To avoid performance instability and assure longer lifetime of the cells, no Li^+ ^cations were added in the above mixture [15].

### Solar cell assembly and measurements

To construct the DSCs, opaque TiO_2 _films (about 12-15 μm thick, measured with a profilometer) were deposited by doctor-blading a paste of Degussa P25 powder on transparent conductive glass substrates (Pilkington, Active glass, 15 Ohm/square) and sintered at 450°C for 60 min in air [18]. For optimum photovoltaic performance studies, a second layer of large scattering particles (Ti-Nanoxide 300, Solaronix) was deposited on top of the first one and then the double-layered films were post-treated with TiCl_4_. After final calcination at 550°C for 1 h, sensitization was achieved by immersing the photoelectrodes in a solution of standard N719 dye (Dyesol Ltd.). Open (non-sealed) DSCs were fabricated by putting a small drop of the novel liquid electrolytes onto the sensitized photoelectrode and simply sandwiching the Pt counter electrode against the first electrode. The techniques used for the photoelectrochemical investigation of the cells (*J*-*V *characteristics, Intensity Modulated Voltage Spectroscopy and Intensity Modulated Photocurrent Spectroscopy) are described in detail in [18]. It should be noted that a good batch of DSCs was tested and a mean value for the obtained results has been taken into account. Low statistical errors (see tables) validate the repeatability of the measurements and justify the representative outcome for our analysis.

## Results and discussion

### Effects of iodine concentration in the electrolyte in the presence of TBP or NMBI additives

**Figure 1:**
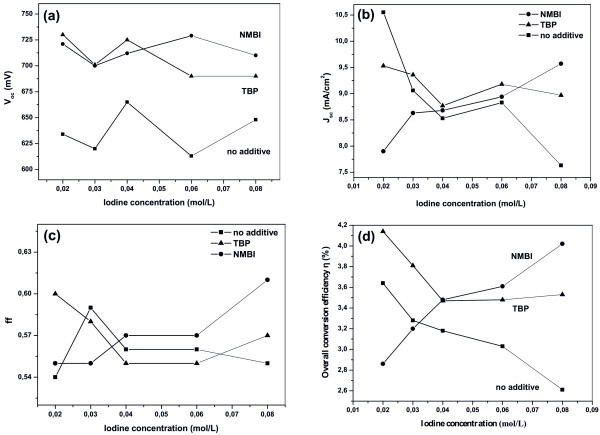
**Variation of the DSCs characteristic parameters as a function of the iodine concentration in the electrolyte**. (**a**) open-circuit potential (*V*_oc_), (**b**) short-circuit photocurrent (*J_sc_*), (**c**) fill factor (*ff*), and (**d**) overall power conversion efficiency (*η*). Squares correspond to the absence of additive, whereas triangles and circles to the TBP and NMBI addition, respectively.

After incorporation of the electrolytes inside the cells, DSCs were assembled and their *J*-*V *characteristics were determined. In a first attempt, the effect of the iodine concentration in the electrolyte (without any additive) was investigated. Table S1 in Additional file 1 presents the photovoltaic parameters obtained from the *J*-*V *characteristic curves (Figure S1 in Additional file 1) of the PMII-I_2_-PC electrolytes, while Figure 1 presents analytically the dependence of the cell parameters (*V*_oc_, ff, *J*_sc _and η) on the iodine concentration. As we can see from Figure 1b, the short-circuit photocurrent density (*J*_sc_) decreases systematically as the [I_2_] increases, in agreement with literature results [19]. The above behaviour can be understood by assuming that whereas a specific critical level of I_3_^- ^is necessary for cell functioning, further increase of [I_2_] (or of the produced [I_3_^-^]), increases recombination at short-circuit conditions, thus reducing *J*_sc_[20].

When TBP was added in the PMII redox electrolyte (the *J*-*V *characteristic curves are shown in Figure S2 (Additional file 2), while Table S2 in Additional file 2 summarizes the photovoltaic parameters in detail), the *V*_oc _of the corresponding cells was increased under all circumstances (Figure 1a). Most importantly, the tendency that was previously observed regarding the systematic reduction of *J*_sc _with increasing [I_2_] is generally preserved (Figure 1b). Again, the *J*_sc _is significantly decreased (at least until 0.04 M), while a further increase of the iodine content does not significantly modify the observed *J*_sc_.

To make a comparison between electrolytes containing different additives which play an identical effective role in DSCs, NMBI was now added in the pristine (no TBP) PMII electrolytes. The *J*-*V *characteristics of cells with NMBI are depicted in Figure S3 (Additional file 3), while Table S3 in Additional file 3 presents the photovoltaic parameters derived from the current-potential curves. By comparing the *V*_oc _values in Figure 1a, it is thus evident that, like in the case of TBP, *V*_oc _is increased for all [I_2_] by 45-115 mV, in line with literature [11,21]. On the other hand, concerning the *J*_sc _variation with [I_2_], the behaviour (previously met with the no additives and TBP-based systems) changes now radically. The results reveal that for electrolytes containing [I_2_] < 0.04 M, the addition of NMBI results in a remarkable decrease of *J*_sc _(Figure 1b), in agreement with relevant literature results [22]. However, for [I_2_] higher than 0.04 M, the *J*_sc _was substantially increased and finally the highest photocurrent (9.57 mA/cm^2^) was obtained using the electrolyte with the highest concentration of iodine (0.08 M).

All the above results are quantified through Figure 1d that presents the overall power conversion efficiencies of the DSCs using electrolytes with I_2 _in varying concentrations, with and without additives. By comparing Figure 1b, d, it is evident, then, that *J*_sc _is the main factor determining the photovoltaic efficiency as we can see that both *J*_sc _and η curves follow very similar trends. However, from Figure 1a, c it is clear that the other two parameters, *V*_oc _and ff, are varied in a non-systematic way, when changing [I_2_]. Another significant feature is that the highest efficiencies are obtained at [I_2_] = 0.02 M when no additive is used in the electrolyte (η = 3.64%) or when TBP is added (η = 4.14%), Figure 1d. On the contrary, the highest efficiency for NMBI is obtained at very high iodine concentrations ([I_2_] = 0.08 M). This value is very similar to the one measured with the TBP additive (η = 4.12%), but the relative efficiency increase is now spectacular (since η of the DSC at 0.08 M without NMBI is only 2.61%), leading us to the conclusion that the presence of NMBI additive smooths the iodine concentration effects.

The behaviour exhibited by the TBP-based versus the NMBI-based electrolyte (both are electron donating nitrogen heterocycles) can be easily understood if someone takes into account the differences in chemical affinity of the two organic additives with respect to the iodine [23-25], fact that significantly affects their adsorption on the TiO_2 _surface.

### GuNCS addition in the optimum electrolytes and electron dynamics analysis of the DSCs

**Figure 2:**
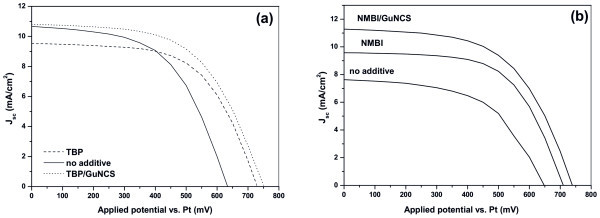
**Additive effects on *J*-*V *characteristic curves for DSCs using optimum electrolytes**. (**a**) PMII-0.02 M I_2_-PC electrolyte without additive as well as in the presence of TBP additive and TBP/GuNCS co-additives. (**b**) PMII-0.08 M I_2_-PC electrolyte without additive as well as in the presence of NMBI additive and NMBI/GuNCS co-additives.

**Figure 3:**
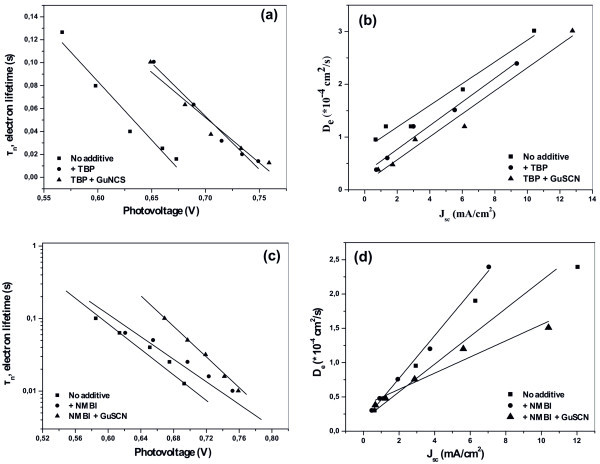
**Additive effects on electron recombination and transport properties for DSCs using optimum electrolytes**. (**a**) Electron lifetimes (*τ_η_*) versus photovoltage (*V_oc_*) and (**b**) electron diffusion coefficients (*D_e_*) versus the short-circuit photocurrent (*J_sc_*), for PMII-0.02 M I_2_-PC electrolyte without additive as well as in the presence of TBP additive and TBP/GuNCS co-additives. (**c**) Electron lifetimes (*τ_η_*) versus *V*_oc_, and (d) electron diffusion coefficients (*D_e_*) versus *J*_sc_, for PMII-0.08 M I_2_-PC electrolytes without additive, with NMBI and with NMBI-GuNCS co-additives.

The DSCs presenting the optimum efficiency have been then used as reference cells for further experiments. To this end, GuNCS was added in the electrolytes already containing the TBP or NMBI additives to significantly increase the *J*_sc _(and slightly the *V*_oc_) of the cells. The *J*-*V *curves of the cells were drawn in Figure 2 while a comparative evaluation of the results is summarized on Tables 1 and 2. Additionally, Intensity Modulated Voltage Spectroscopy (IMVS) and Intensity Modulated Photocurrent Spectroscopy (IMPS) were used to extract valuable information about recombination kinetics under open-circuit and charge transport properties at short-circuit conditions, respectively [26]. Figure 3a, c presents the plots of the electron lifetime (τ_n_) versus the photovoltage while Figure 3b, d displays the plots of the electron diffusion coefficients (*D*_e-_) versus the photocurrent.

**Table 1:** Performance parameters of the DSCs using PMII-0.02 M I_2_-PC electrolytes without additive, with TBP and with TBP-GuNCS as co-additives.

Additives	*J*_sc _(mA/cm^2^)	*V*_oc _(mV)	ff	η(%)
No	10.55 ± 0.24	634 ± 0	0.54 ± 0.01	3.64 ± 0.02
TBP	9.53 ± 0.08	730 ± 28	0.60 ± 0.01	4.14 ± 0.24
TBP + GuNCS	10.76 ± 0.19	747 ± 8	0.57 ± 0.02	4.59 ± 0.19

**Table 2:** Performance parameters of the DSCs using PMII-0.08 M I_2_-PC electrolytes without additive, with NMBI and with NMBI-GuNCS as co-additives.

Additives	*J*_sc _(mA/cm^2^)	*V*_oc _(mV)	ff	η (%)
No	7.63 ± 0.80	648 ± 24	0.55 ± 0.00	2.61 ± 0.04
NMBI	9.57 ± 0.64	710 ± 9	0.61 ± 0.03	4.12 ± 0.16
NMBI ± GuNCS	11.28 ± 0.07	739 ± 4	0.57 ± 0.01	4.71 ± 0.00

Taking a close look at the results of Table 1, it appears that the addition of the TBP leads to *V*_oc _increase due to the synergistic effects of the negative *E*_c _shift along with the increase of electrons lifetimes (Figure 3a), in perfect agreement with the literature [10]. Simultaneously, TBP decreases *J*_sc_, due to the *E*_c _shift (which also entails a smaller driving force for electron injection from the excited dye [10]) as well as due to the reduced electron diffusion coefficients (Figure 3b). The extra addition of GuNCS in the TBP-based electrolytes increased further the gained *V*_oc _(by only 17 mV in perfect agreement with the 20 mV increase observed by Kopidakis et al. in [8]), whereas the recombination kinetics at open-circuit are not affected (no variation of the electron lifetimes, Figure 3a). However, and most importantly, the addition of GuNCS restored the values of *J*_sc _by positively shifting the *E*_c _of TiO_2 _(that consequently increases the electrons injection efficiency [12]), and this happens despite the fact that the *D*_e- _were further decreased (Figure 3b). The *V*_oc _invariance in conjunction with the large *J*_sc _increase can be only explained by a large *E*_c _shift towards positive potentials giving rise to a large injection efficiency under both open and short-circuit conditions or, most unlikely, by a Fermi level pinning.

On the contrary, the addition of NMBI in the electrolytes causes different effects. Figure 2b (and more clearly Table 2) proves that the addition of NMBI increases both *V*_oc _and *J*_sc_; the increase of *J*_sc _is due to the enhanced electron diffusion coefficients (Figure 3d) and despite the fact that the conduction band edge of TiO_2 _moves to more negative potentials [27]. On the other hand, *V*_oc _also increases due to the reduction of the electrons back-reaction and this is expressed in increased values of electron lifetime (Figure 3c), in agreement with [11]. Furthermore, the addition of GuNCS in the NMBI-based electrolytes increased further the gained *V*_oc _(by 19 mV in line with the *V*_oc _increase observed in the TBP/GuNCS electrolytes), due to the additional increase of the electron lifetimes; the reduction of recombination seems to be strong enough to determine the *V*_oc _of the cell (which is slightly increased, instead of an anticipated large decrease due to *E*_c _shift towards positive potentials). The above results are in great accordance with Zhang et al. [12]. Most considerably, GuNCS increased the *J*_sc _values (from 9.5 up to 11.2 mA/cm^2^) despite the decreased *D*_e- _(Figure 3d); it seems that the *E*_c _shift is the main parameter (and not electron transport kinetics) determining the overall photocurrent delivered by the cell.

It was thus concluded that although some additives (TBP and NMBI) have similar chemical properties, both shift the *E*_c _towards negative values and reduce recombination but, finally, they act in a total different way in IL-based liquid electrolytes dissolved in PC solvent, affecting also electron transport kinetics and thus influencing *J*_sc_. GuNCS was added in the electrolyte to compensate the *E*_c _shifts and increase the photocurrent (while also slightly increase *V*_oc _by reducing recombination). Even if this was indeed the result, GuNCS again acts in a different manner in each studied system (affecting recombination at open-circuit only in the NMBI system). The overall efficiencies with TBP-GuNCS and NMBI-GuNCS electrolytes was very similar reaching values up to 4.59 and 4.71%, respectively.

### Optimization of the cells efficiency

**Figure 4:**
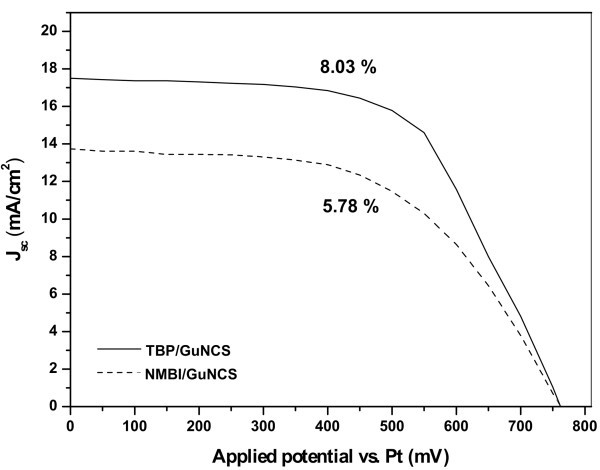
***I*-*V *curves for optimum DSCs (using optimum multiple layered TiO_2 _films) and the TBP or NMBI addition into the PMII-I_2_-GuNCS-PC electrolytes**.

The above efficiencies were determined using non-optimized DSCs because we decided to stay in a system as simple as possible, in order to clearly discriminate the additive effects. Thus in a last step, we have used optimized TiO_2 _films electrodes (with a three layer stratification of 22 μm in total thickness, incorporating Degussa P25, scattering layer and titania nanoparticles from TiCl_4 _treatment) with enhanced electrooptical properties to increase the above efficiencies and find out, at the end, what is the maximum gain for a solar cell when using the two additives together in the electrolyte. From Figure 4, depicting the two *I*-*V *curves with optimum DSCs, one can infer that the electrolyte with NMBI-GuNCS gives a maximum efficiency of 5.78% (with *J*_sc _= 13.7 mA/cm^2^, *V*_oc _= 762 mV and ff = 0.55). However, a much higher efficiency of 8.03%, resulting from much higher *J*_sc _of the order of 17.5 mA/cm^2 ^(*V*_oc _= 763 mV and ff = 0.60), is achieved when TBP and GuNCS are simultaneously incorporated inside the electrolyte. The above results are in great accordance with the optimum composition of electrolytes found in recent literature dealing with state-of-the-art cells [14]. In the case of the optimized DSCs, the much better performance of the TBP-based electrolyte (with respect to the cells using NMBI-based electrolyte) is mainly due to the particular stratification of the composite electrode. In fact, the adsorption behaviour of the TBP and NMBI additives on TiO_2 _could be differentiated by the small-sized titania nanoparticles (produced following the TiCl_4 _treatment), thus affecting in a different way the electron injection/recombination dynamics.

## Conclusions

In this work, we have studied and compared the influence of additives (TBP, NMBI and GuNCS) in lithium-free ionic liquid-based electrolytes containing the I^-^/I_3_^- ^redox couple dissolved in propylene carbonate. Despite the fact that generally TBP and NMBI work similarly as proposed in related literature by increasing *V*_oc_, NMBI was only found to smooth the high iodine concentration effects (that would normally increase recombination at short-circuit) by also simultaneously increasing *J*_sc_. To this end, TBP enhanced the initial efficiency under [I_2_] = 0.02 M from 3.5 up to 4.1%, while NMBI boosted the corresponding initial efficiency at [I_2_] = 0.08 M from 2.5 to 4.1%. Further addition of guanidinium cations in the above systems increased the efficiencies by another 0.5-0.7% (by increasing both *V*_oc _and *J*_sc_); however, again the GuNCS acts differently in each system, affecting recombination at open-circuit only in the NMBI-based electrolyte. Finally, TiO_2 _films of optimum stratification have shown efficiencies of 5.8% with NMBI-GuNCS and up to 8.0% with TBP-GuNCS co-additives, respectively.

## Abbreviations

DSCs: dye-sensitized solar cells; GuNCS: guanidinium thiocyanate; IMVS: Intensity Modulated Voltage Spectroscopy; IMPS: Intensity Modulated Photocurrent Spectroscopy; IL: ionic liquid; NMBI: *N*-methylbenzimidazole; PMII: 1-methyl-3-propylimidazolium iodide; PC: propylene carbonate; TBP: 4-*tert*-butylpyridine.

## Competing interests

The authors declare that they have no competing interests.

## Authors' contributions

TS participated in the design and implementation of the work and help to draft the manuscript. ER carried out the realization of the experiments. CSK have been involved in revising the manuscript critically for important intellectual content. PF conceived the study, participated in its design and coordination, and helped to draft and finalize the manuscript. All authors read and approved the final manuscript.

## Supplementary Material

Additional file 1 **Figure S1**. Current density-voltage (*J*-*V*) characteristics of the DSCs using the PMII-I_2_-PC electrolytes with varying iodine concentration. Table S1. Performance parameters of solar cells using the PMII-I_2 _-PC electrolytes with varying I_2 _concentration. Click here for file

Additional file 2 **Figure S2**. *J*-*V *curves of the DSCs using the PMII-I_2_-TBP-PC electrolytes with varying iodine concentration. Table S2. Performance parameters of the DSCs using the PMII-I_2_-TBP-PC electrolytes with varying I_2 _concentration. Click here for file

Additional file 3 **Figure S3**. *J*-*V *curves of the DSCs using the PMII-I_2_-NMBI-PC electrolytes with varying iodine concentration. Table S3. Performance parameters of the DSCs using the PMII-I_2_-NMBI-PC electrolytes with varying I_2 _concentration. Click here for file
