# Supplementary material for: Influence of electrolyte co-additives on the performance of dye-sensitized solar cells
Source: Nanoscale Res Lett. 2011 Apr 7;6(1):307. doi: 10.1186/1556-276X-6-307 (PMC3211393; doi:10.1186/1556-276X-6-307)
Supplement: Additional file 1 — Figure S1. Current density-voltage (J-V) characteristics of the DSCs using the PMII-I2-PC electrolytes with varying iodine concentration. Table S1. Performance parameters of solar cells using the PMII-I2 -PC electrolytes with varying I2 concentration. [file 1556-276X-6-307-S1.DOC]

### Additional file 1

**Fig. S1** Current density-Voltage (J-V) characteristics of the dye-sensitized solar cells using the PMII-I_2_-PC electrolytes with varying iodine concentration.

**Table S1.** Performance parameters of solar cells using the PMII-I_2_ -PC electrolytes with varying I_2_ concentration.

| [I_2_] | J_sc_ (mA/cm^2^) | V_oc_ (mV) | ff | η (%) |
| --- | --- | --- | --- | --- |
| 0.02M | 10.55±0.24 | 634±0 | 0.54±0.01 | 3.64±0.02 |
| 0.03M | 9.06±0.71 | 620±6 | 0.59±0.02 | 3.28±0.17 |
| 0.04M | 8.53±0.39 | 665±17 | 0.56±0.00 | 3.18±0.06 |
| 0.06M | 8.83±0.38 | 613±3 | 0.56±0.06 | 3.03±0.19 |
| 0.08M | 7.63±0.80 | 648±24 | 0.55±0.00 | 2.61±0.04 |
