# Supplementary material for: Influence of electrolyte co-additives on the performance of dye-sensitized solar cells
Source: Nanoscale Res Lett. 2011 Apr 7;6(1):307. doi: 10.1186/1556-276X-6-307 (PMC3211393; doi:10.1186/1556-276X-6-307)
Supplement: Additional file 2 — Figure S2. J-V curves of the DSCs using the PMII-I2-TBP-PC electrolytes with varying iodine concentration. Table S2. Performance parameters of the DSCs using the PMII-I2-TBP-PC electrolytes with varying I2 concentration. [file 1556-276X-6-307-S2.DOC]

### Additional file 2

**Fig. S2** J-V curves of the DSCs using the PMII-I_2_-TBP-PC electrolytes with varying iodine concentration.

**Table S2.** Performance parameters of the DSCs using the PMII-I_2_-TBP-PC electrolytes with varying I_2_ concentration.

| [I_2_ ] | J_sc_ (mA/cm^2^) | V_oc_ (m V) | ff | η (%) |
| --- | --- | --- | --- | --- |
| 0.02 | 9.53±0.08 | 730±28 | 0.60±0.01 | 4.14±0.24 |
| 0.03 | 9.36±0.27 | 701±3 | 0.58±0.00 | 3.81±0.08 |
| 0.04 | 8.77±0.17 | 725±7 | 0.55±0.01 | 3.47±0.01 |
| 0.06 | 9.18±0.05 | 690±4 | 0.55±0.03 | 3.48±0.15 |
| 0.08 | 8.97±0.44 | 690±0 | 0.57±0.06 | 3.53±0.25 |
