# Supplementary material for: Influence of electrolyte co-additives on the performance of dye-sensitized solar cells
Source: Nanoscale Res Lett. 2011 Apr 7;6(1):307. doi: 10.1186/1556-276X-6-307 (PMC3211393; doi:10.1186/1556-276X-6-307)
Supplement: Additional file 3 — Figure S3. J-V curves of the DSCs using the PMII-I2-NMBI-PC electrolytes with varying iodine concentration. Table S3. Performance parameters of the DSCs using the PMII-I2-NMBI-PC electrolytes with varying I2 concentration. [file 1556-276X-6-307-S3.DOC]

### Additional file 3

**Fig S3.** J-V curves of the DSCs using the PMII-I_2_-NMBI-PC electrolytes with varying iodine concentration.

**Table S3.** Performance parameters of the DSCs using the PMII-I_2_-NMBI-PC electrolytes with varying I_2_ concentration.

| [I_2_] | J_sc_ (mA/cm^2^) | V_oc_ (m V) | FF | η (%) |
| --- | --- | --- | --- | --- |
| 0.02M | 7.90±1.44 | 721±41 | 0.55±0.07 | 3.10±0.34 |
| 0.03M | 8.63±0.02 | 700±2 | 0.55±0.01 | 3.30±0.05 |
| 0.04M | 8.68±0.02 | 712±31 | 0.57±0.01 | 3.52±0.06 |
| 0.06M | 8.94±0.03 | 729±6 | 0.57±0.01 | 3.68±0.10 |
| 0.08M | 9.57±0.64 | 710±9 | 0.61±0.03 | 4.12±0.16 |
| 0.10M | 9.51±0.23 | 703±8 | 0.58±0.05 | 3.84±0.28 |
